# Characterization of BAFF and APRIL subfamily receptors in rainbow trout *(Oncorhynchus mykiss)*. Potential role of the BAFF / APRIL axis in the pathogenesis of proliferative kidney disease

**DOI:** 10.1371/journal.pone.0174249

**Published:** 2017-03-21

**Authors:** Aitor G. Granja, Jason W. Holland, Jaime Pignatelli, Christopher J. Secombes, Carolina Tafalla

**Affiliations:** 1 Centro de Investigación en Sanidad Animal (CISA-INIA). Valdeolmos (Madrid), Spain; 2 Scottish Fish Immunology Research Centre, University of Aberdeen, Aberdeen, United Kingdom; Institut Cochin, FRANCE

## Abstract

Proliferative kidney disease (PKD) is a parasitic infection of salmonid fish characterized by hyper-secretion of immunoglobulins in response to the presence of the myxozoan parasite, *Tetracapsuloides bryosalmonae*. In this context, we hypothesized that the BAFF/APRIL axis, known to play a major role in B cell differentiation and survival in mammals, could be affected by the parasite and consequently be involved in the apparent shift in normal B cell activity. To regulate B cell activity, BAFF and APRIL bind to transmembrane activator and calcium modulator and cyclophilin ligand interactor (TACI) and B cell maturation antigen (BCMA), whereas BAFF also binds to BAFF receptor (BAFF-R). In teleost fish, although some BAFF and APRIL sequences have been reported, their receptors have not been identified. Thus, as a first step in the current work, we have identified homologues to mammalian TACI, BCMA and BAFF-R in rainbow trout (*Oncorhynchus mykiss*), that constitute the first report of BAFF and APRIL receptor sequences in fish. Subsequently we studied the transcriptional modulation of BAFF, APRIL, and the fish-specific related cytokine, BALM and their putative receptors in fish naturally exposed to *T*. *bryosalmonae*. Finally, to gain further insights on the functional role that these cytokines play during the course of PKD, we have studied their effect on the survival of kidney IgM^+^ B cells and on immunoglobulin transcription. Our results support the premise that the BAFF / APRIL axis could play an important role during PKD, which may open the possibility of new therapeutic treatments against the disease.

## Introduction

The tumor necrosis factor (TNF) superfamilies of ligands and receptors (TNFRs) play a key role in development, tissue homeostasis and the initiation of innate and adaptive immune responses [1]. With respect to the latter, most TNF superfamily members play essential roles in different aspects of B cell biology, from their development in hematopoietic tissues, to their maturation in peripheral tissues and their differentiation into memory or plasma cells [2]. Interestingly, cooperative signaling via TNF ligands and receptors was first seen within the CD40-CD40 ligand (CD40L) interaction, that promotes the proliferation of antigen-activated B cells through the expression of CD40L by CD4^+^ T helper (Th) cells [3]. B cells do not only receive co-stimulating signals from Th cells but can also receive them from phagocytes such as macrophages, dendritic cells (DCs) and granulocytes that secrete cytokines in response to pro-inflammatory stimuli or after recognition of invariant pathogenic patterns. BAFF (B cell activating factor) and APRIL (a proliferation inducing ligand), are two of the main cytokines produced by innate immune cells to co-stimulate B cells [4]. As with many of the TNF family ligands, BAFF and APRIL are produced as transmembrane proteins that are proteolytically cleaved at a furin protease site and released in a soluble form [5]. Both cytokines regulate B cell survival, proliferation and differentiation, especially during innate immune responses [6].

Ligand–receptor interactions within the BAFF–APRIL subfamily of TNF ligands are both redundant and specific. BAFF binds to the BAFF receptor (BAFF-R, also known as TNFR13C), to transmembrane activator and CAML interactor (TACI, also known as TNFR13B) and, with lower affinity, to B-cell maturation antigen (BCMA, also known as TNFR17), whereas APRIL binds to TACI and BCMA (reviewed in [7]). APRIL also interacts with the polysaccharide side chains of heparan sulfate proteoglycans (HSPGs), structurally unrelated to TNF receptors [8].

In fish, many different TNF superfamily members have been identified in diverse fish species. Regarding the BAFF and APRIL subfamily of ligands and receptors, BAFF sequences have been reported recently in different teleosts including zebrafish (*Danio rerio*) [9], mefugu (*Takifugu obscurus*) [10], Japanese sea perch (*Lateolabrax japonicus*) [11], grass carp (*Ctenopharyngodon idella*) [12], yellow grouper (*Epinephelus awoara*) [13], miiuy croaker (*Miichthys miiuy*) [14], tongue sole (*Cynoglossus semilaevis*) [15], Nile tilapia (*Oreochromis niloticus*) [16], rock bream (*Oplegnathus fasciatus*) [17], rainbow trout (*Oncorhynchus mykiss*) [18], rohu (*Labeo rohita*) [19] and cartilaginous fish such as white-spotted catshark (*Chiloscyllium plagiosum*) [20], spiny dogfish (*Squalus acanthias*) [21] and small-spotted catshark (*Scyliorhinus canicula*) [22]. APRIL sequences, on the other hand, have only been identified in channel catfish (*Ictalurus punctatus*), Atlantic salmon (*Salmo salar*) and rainbow trout [18, 22]. Interestingly, in species such as rainbow trout, a cytokine designated as a BAFF and APRIL-like molecule (BALM) has also been reported [18]. Given the absence of BALM sequences in tetrapods and its recent discovery in lampreys, BALM has been postulated as an ancestral homolog of BAFF and APRIL [23]. As for the receptors of BAFF and APRIL, no BAFF-R, BCMA or TACI sequences have been reported in fish to date. Thus, the role played by these cytokines and their receptors during the fish immune response is currently unknown.

Proliferative Kidney Disease (PKD), caused by the myxozoan parasite *Tetracapsuloides bryosalmonae*, is a slow progressive disease of major economic importance to salmonid aquaculture [24]. Parasite spores, released from infected freshwater bryozoans, gain entry into the fish host vascular system via the gills [25] and migrate to different organs, with the posterior kidney being the main focus of parasite development and proliferation [24]. In teleosts, the head kidney is the main hematopoietic tissue where B cells develop and most proliferating B cell precursors are found. The posterior kidney, on the other hand, houses significant populations of partially activated B cells and plasmablasts [26]. Extrasporogonic proliferation and development of *T*. *bryosalmonae* in the kidney interstitial tissue provokes chronic immunopathology characterized by a lymphocytic hyperplasia, formation of granulomatous lesions, renal atrophy, and hyper-secretion of immunoglobulins [24, 27]. Furthermore, recent transcriptional analysis of the kidney in naturally infected fish with different degrees of PKD also pointed to dysregulation of B cell activity in response to the parasite [28]. In this context, trout BAFF / APRIL ligands and receptors could be implicated in the pathogenesis of this disease. Thus, in this study we have sequenced and characterized rainbow trout BAFF-R, BCMA and TACI and, along with their potential ligands, studied their transcriptional modulation in the kidneys of fish naturally infected by the parasite. Additionally, we have studied the effect of recombinant BAFF, APRIL and BALM on survival of IgM^+^ B cells and immunoglobulin transcription in the kidney. Our results reveal a potential role of the BAFF / APRIL axis during the course of PKD pathogenesis that may open the door to potential anti-parasitic treatments, which are discussed.

## Materials and methods

### Identification of BAFF receptor sequences

Murine and human BAFF-R protein sequences were used as tBLASTn queries against rainbow trout (*Oncorhynchus mykiss*) expressed sequence tag (EST) databases within the National Center for Biotechnology Information database (http://blast.ncbi.nlm.nih.gov/Blast.cgi). A rainbow trout EST encoding a BAFF-R-like sequence was identified (accession number CA381223.1). The sequence lacked a stop codon, therefore 3' RACE (Rapid Amplification of cDNA Ends) (Invitrogen) was used to obtain the full length sequence from a mixed tissue cDNA sample with specific primers (Table 1). Sequences corresponding to Atlantic salmon (*Salmo salar*) BCMA and TACI homologues were retrieved from GenBank (accession numbers XM_014178965 and XM_014178939.1 respectively). Primers designed from the salmon sequences (Table 1) were used to generate complete and incomplete rainbow trout BCMA and TACI transcripts from a mixed tissue cDNA sample respectively. 3’RACE was again performed to obtain the full coding sequence and 3´UTR of rainbow trout TACI (Table 1).

**Table 1:** Primers used for PCR cloning and real-time PCR analysis of gene expression

Gene	Primer name	Primer sequence (5´-3´)	Application
TACI	SS-TACI-F	GCTCAGTGTCACTGCATGAGAGGC	Cloning
	SS-TACI-R	ACACGTCTCTGTGGGGCTGG	Cloning
	TACI-3-RACE	GTGTCTGGCTCTGCTGCTGTTGACC	3´RACE
	TACI-3-RACE-nest	GTGCTGCTGAGGAGGGCCAGTGCC	3´RACE
	RT-TACI-F	GCATCGAGTACTGTGCTTCTCTAGG	Real time PCR
	RT-TACI-F	AAGTCAGGCTGTTGGGTCTTACATT	Real time PCR
BCMA	SS-BCMA-F	ATGTCAGAAGGACAGTGTGGACTGG	Cloning
	SS-BCMA-R	CTGTGTGGTTAAAGATCTGTACTGTCTTGG	Cloning
	RT-BCMA-F	ATGTCAGAAGGACAGTGTGGACTGG	Real time PCR
	RT-BCMA-R	CGGCTCTGGGGCTTTGCTCT	Real time PCR
BAFF-R	BAFFR-3-RACE-	CCTCGGTGTGTGGCTCTCTTGGGG	3´RACE
	BAFFR-3-RACE-nest	GGGAGCATTGGATCCCACTTCCTGC	3´RACE
	RT-BAFFR-F	TGTCTGGATATCAATGGTCGTCATA	Real time PCR
	RT-BAFFR-R	CTTTAGCTGGAGGGTTAAGTCTTGC	Real time PCR
BAFF	RT-BAFF-F	ATGTTTGATGCTTATTCTGGCAGGT	Real time PCR
	RT-BAFF-R	TGGGACTGTGTCTTGACTGTGTGTA	Real time PCR
APRIL	RT-APRIL-F	CACAGACATACACAATGGAATGGAA	Real time PCR
	RT-APRIL-R	TGTGATGACAGAGGAACAAGATGAA	Real time PCR
BALM	RT-BALM-F	TGGAGGTACAGTAGTTCAGCAGTCG	Real time PCR
	RT-BALM-R	ACTATCCAAGGAATCACCGTCACAT	Real time PCR
IgMtotal	RT-IgMtotal-F	TGCGTGTTTGAGAACAAAGC	Real time PCR
	RT-IgMtotal-R	GACGGCTCGATGATCGTAAT	Real time PCR
IgMsec	RT-IgMsec-F	CCTTAACCAGCCGAAAGGG	Real time PCR
	RT-IgMsec-R	TGAGGTTCTATCAATGGTTCTC	Real time PCR
IgT	RT-IgTtotal-F	AACATCACCTGGCACATCAA	Real time PCR
	RT-IgTtotal-R	TTCAGGTTGCCCTTTGATTC	Real time PCR
IgD	RT-IgDtotal-F	AGCTACATGGGAGTCAGTCAACT	Real time PCR
	RT-IgDtotal-R	CTTCGATCCTACCTCCAGTTCCT	Real time PCR
EF-1α	RT-EF1α-F	GATCCAGAAGGAGGTCACCA	Real time PCR
	RT-EF1α-R	TTACGTTCGACCTTCCATCC	Real time PCR

Protein analysis was performed using the ExPASy Molecular Biology server (http://us.expasy.org). Similarity searches were performed by BLASTP analysis (http://blast.ncbi.nlm.nih.gov/Blast.cgi) and phylogenetic analyses performed using MegAlign Software (DNAstar Inc., Madison, USA) and the ClustalW algorithm. Bootstrapped phylogenetic trees (x 1000 replicates) were built using the neighbor-joining method. The TMHMM program Server (v. 2.0) (http://www.cbs.dtu.dk/services/TMHMM/) was used to predict protein structure.

### Fish maintenance

Female rainbow trout of approximately 90–100 g were obtained from Centro de Acuicultura El Molino (Madrid, Spain) and maintained at the animal facilities of the Centro de Investigación en Sanidad Animal (CISA-INIA, Spain) in a re-circulating water system at 16°C under a 12:12 h light dark photoperiod. Fish were fed twice a day with a commercial diet (Skretting, Spain). Prior to any experimental procedure, fish were acclimatized to laboratory conditions for at least 2 weeks. The experiments described comply with the Guidelines of the European Union Council (2010/63/EU) for the use of laboratory animals and were approved by the Ethics committee from the Instituto Nacional de Investigación y Tecnología Agraria y Alimentaria (INIA; Code CEEA 2011/044; Permit Number: PROEX 309/14). Fish were anaesthetized with benzocaine (Sigma) prior to being killed, following the recommendations from Zhal *et al*. [29]. All efforts were made to minimize suffering.

### Tissue collection

Blood was extracted from the caudal vein of freshly killed rainbow trout, using a heparinized needle/syringe. Transcardial perfusion using teleost Ringer solution pH 7.4 containing 0.1% procaine was undertaken to remove all blood from fish tissues. Spleen, head kidney, intestine, gills, brain, liver and muscle samples were then collected and placed in Trizol [30, 31]. Single cell suspensions from spleen, head kidney and gills were prepared by pushing the tissues through 100 μm nylon cell strainers (BD Biosciences). Intestine cell suspensions were also prepared. Samples corresponding to the hindgut were opened lengthwise, washed in PBS and cut into small pieces. Prior to cell extraction, pieces of gut tissue were agitated for 30 min at 4°C in L-15 medium containing 100 I.U./ml penicillin, 100 ug/ml streptomycin (P/S) and 5% fetal calf serum (FCS), followed by agitation for 30 min in PBS containing 1 mM EDTA and 1 mM DTT. Tissue digestion was performed using 0.15 mg/ml collagenase type IV from *Clostridium histolyticum* (Sigma) in L-15 for 1.5 h at 20°C. All cell suspensions were placed onto 30 / 51% Percoll (GE Healthcare) density gradients and centrifuged at 500 x *g* for 30 min at 4°C. Cells at the interface were collected and washed twice in L-15 medium containing 5% FCS.

### Gene expression in fish tissues

DNase I-treated total RNA was prepared from tissue samples using a combination of Trizol (Invitrogen) and an RNAeasy Mini kit (Qiagen) as described previously [32]. Total RNA was eluted from the columns in RNase-free water, quantified using a Nanodrop 1000 spectrophotometer (Thermo Scientific) and stored at -80°C. For each sample, 2 μg of total RNA was reverse transcribed using Bioscript reverse transcriptase (Bioline Reagents Ltd) primed with oligo (dT)_12-18_ (0.5 μg/ ml), following the manufacturer´s instructions. cDNA was diluted in nuclease-free water and stored at -20°C.

To evaluate the levels of transcription of the different genes, real-time PCR was performed in a LightCycler 96 System instrument (Roche) using FastStart Essential DNA Green Master reagents (Roche) and specific primers (shown in Table 1). The efficiency of the amplification was determined for each primer pair using serial 10 fold dilutions of pooled cDNA, and only primer pairs with efficiencies between 1.95 and 2 were used. Each sample was measured in duplicate under the following conditions: 10 min at 95°C, followed by 40 amplification cycles (30 s at 95°C and 1 min at 60°C). The expression of individual genes was normalized to that of trout EF-1α and expression levels calculated using the 2^-ΔCt^ method, where ΔCt is determined by subtracting the EF-1α value from the target Ct as described previously [33, 34]. Negative controls with no template were included in all experiments. A melting curve for each PCR was determined by reading fluorescence every degree between 60°C and 95°C to ensure only a single product had been amplified.

### Gene expression at early life stages

To investigate if TACI, BCMA and BAFF-R are expressed at early life stages, eyed eggs at different degree days (DD) post-fertilization (~306 DD, ~354 DD, ~402 DD), immediate post hatch fry (hatch, ~450 DD), pre-first feeding fry (PFF, ~562 DD), fry at the stage of full disappearance of the yolk sac (first feeding, FF, ~674 DD), and fry three weeks following first feeding (Fry, 786 DD) were sampled. The fish were maintained at 16°C in recirculated freshwater. Total RNA was extracted and cDNA prepared for real-time PCR analysis from eggs or whole fry using a combination of Trizol (Invitrogen) and an RNAeasy Mini kit (Qiagen) as described above.

### Gene expression in isolated IgM^+^ cells

Leukocyte suspensions were incubated for 30 min on ice with an anti-trout IgM mAb (1.14) [35] coupled to phycoerythrin (PE) in staining buffer (PBS containing 1% FCS and 0.5% sodium azide) that prevents cell activation. Following two wash steps, cells were resuspended in FACS buffer and IgM^+^ B cells isolated based on their FSC/SSC profiles (excluding the granulocyte gate) and fluorescence emitted by anti-trout IgM (S1 Fig).

DNase I-treated total RNA was reverse transcribed directly from IgM^+^ and IgM^-^ sorted populations using the Power Sybr Green Cells-to-Ct Kit (Invitrogen) following the manufacturer´s instructions. Real-time PCR was performed using SYBR Green PCR core Reagents (Applied Biosystems) following the manufacturer´s instructions as described previously [31].

### Gene expression in response to PKD infection

Two groups of fish from the same egg source (50–100 g each) were sampled for this study: a parasite-naïve uninfected group and a parasite-naïve group exposed to parasite-infected water, as described previously [28]. Briefly, the sampling of both groups was undertaken when naïve parasite-exposed fish exhibited kidney pathology ranging from early to advanced clinical stages (kidney swelling grades 1 to 3), as determined using the kidney swelling index system devised by Clifton-Hadley and colleagues [36]. All control fish had a kidney swelling grade of 0 whereas the presence of *T*. *bryosalmonae* in parasite-exposed fish was confirmed by histological examination of posterior kidney smears. In all fish sampled, approximately 100 mg of kidney tissue was removed immediately below the dorsal fin, the area of the kidney associated with the onset of clinical disease. Tissue samples were placed into 1 ml of RNA-later (Sigma, ST. Louis, USA), kept at 4°C for 24 h and stored at -80°C prior to RNA extraction and PCR analysis. Total RNA was extracted using TRI-reagent (Sigma) according to the manufacturer’s instructions. Purified RNA was quantified using a Nanodrop spectrophotometer (NanoDrop Technologies, Wilmington, USA) and reverse transcribed into cDNA as described above. To evaluate the levels of transcription of the different genes, real-time PCR was performed using Fast-Start Essential DNA Green Master reagents following the protocol described above.

### Production of recombinant BAFF, APRIL and BALM

To study the effects of rainbow trout BAFF, APRIL and BALM on kidney IgM^+^ B cell survival and immunoglobulin transcription, we produced the three cytokines in *E*. *coli*. To carry this out, the nucleotide sequence corresponding to the extracellular domain of the rainbow trout BAFF sequence (GenBank Accession number DQ218467.1), APRIL (GenBank Accession number EF451543.1) or BALM (GenBank Accession number DQ218469.1) sequences together with an N-terminal 6 x histidine tag were synthesized and subcloned into the E3 expression vector (Abyntek). The recombinant plasmids were transformed into BL21 cells and kanamycin-resistant single positive colonies for each clone were then incubated at 37°C in Luria-Bertani (LB) media. When the OD_600_ reached 0.6, 0.1 mM of isopropyl β-D-thiogalactoside (IPTG, Sigma Aldrich) was added to induce protein production. After 16 h, cells were harvested, lysed by sonication and dissolved using urea. Thereafter, BAFF, APRIL or BALM were obtained through the use of Nickel columns (Sigma Aldrich). The protein-containing fractions were pooled, refolded, filtered through 0.22 μm and resuspended in storage buffer (50 mM Tris-HCl, 150 mM NaCl, 10% glycerol, 0.5 M L-arginine and 2 mM DTT, pH 8.5). Protein concentrations were determined in a BCA protein assay (Thermo Fisher Scientific) and the recombinant rainbow trout cytokines (0.3 mg/ml) aliquoted and stored at -80^°^C until used. An irrelevant protein with a similar molecular weight to that of these recombinant proteins, also bearing an N-terminal His tag was produced in the same conditions (C-His) and was used as a functional control.

### Effect of BAFF, APRIL and BALM on survival and proliferation of kidney IgM^+^ B cells

Kidney leukocytes were incubated in the presence of recombinant rainbow trout BAFF, APRIL or BALM at a final concentration of 3 μg/ml, in the presence of LPS (50 μg/ml) or with media alone (control). After 72 h of incubation, cells were reacted with PE-labeled anti-IgM (1.14) antibody and live IgM^+^ B cells were quantified by flow cytometry. In those cases in which increased IgM^+^ B cell survival was observed, we analyzed cell proliferation to establish whether the cells were actively proliferating in response to the cytokine or only an increased survival was occurring. For this, kidney leukocytes were incubated in the presence of BALM (3 μg/ml), LPS (50 μg/ml) or left unstimulated (control) at 20°C. After 96 h of incubation, 10 μM EdU was added to the cultures for 2 h, and cells were reacted with APC-labeled anti-IgM antibody (1.14), and then treated for subsequent cell proliferation analysis following the manufacturer´s instructions (Click-iT® Plus EdU Alexa Fluor 488 Flow Cytometry Assay kit, Thermo Fisher Scientific). All samples were analyzed on a FACSCalibur flow cytometer (BD Biosciences) equipped with CellQuest Pro software. Analysis was performed with FlowJo 10 (TreeStar)

### Effect of BAFF, APRIL and BALM on immunoglobulin transcription in kidney

Kidney leukocytes were incubated at 20°C in the presence of recombinant rainbow trout BAFF, APRIL or BALM, or the C-His control protein, at a final concentration of 3 μg/ml. After 24 h, total RNA was extracted using TRI-reagent (Sigma) according to the manufacturer’s instructions. Purified RNA was quantified using a Nanodrop spectrophotometer (NanoDrop Technologies, Wilmington, USA) and reverse transcribed into cDNA as described above. The levels of transcription of IgM and IgT were evaluated by real-time PCR using Fast-Start Essential DNA Green Master reagents following the protocol described above.

### Statistics

Data handling, statistical analyses and graphic representation were performed using Office Excel 2010 (Microsoft Corporation) and GraphPad Prism v. 6.0 (GraphPad Software Inc.). Statistical differences between populations were analyzed using a two tailed unpaired Student’s *t* test. Correlations between *T*. *bryosalmonae* (PKD) kidney swelling grade and immune gene expression were assessed by calculating the Pearson product–moment correlation coefficient (r) and considered significant at *P* ≤ 0.05 (using the two tailed Student’s *t* test). Correlation between parameters was represented by a linear regression.

## Results

### Sequence analysis of the rainbow trout BAFF receptor sequences

**Fig 1:**
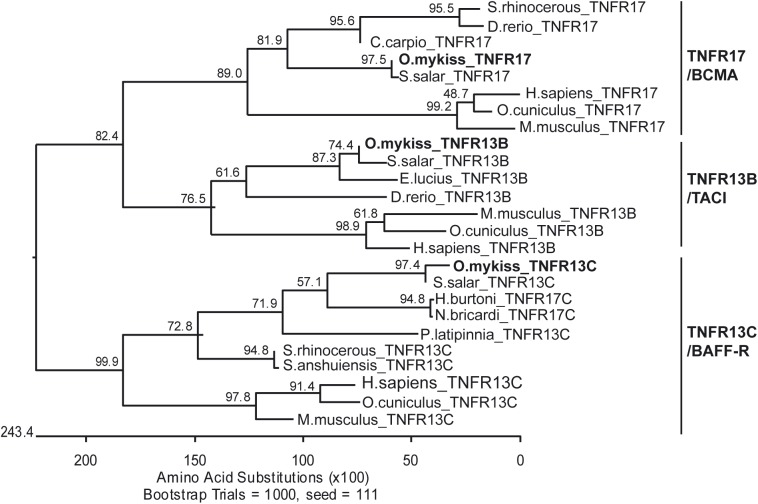
Phylogenetic analysis of rainbow trout BAFF-R, BCMA and TACI. The rooted phylogenetic tree shows the relationship between available sequences and those representing rainbow trout TNFR17 or BCMA, TNFR13B or TACI and TNFR13C or BAFF-R (in bold) with other fish species and with human and other available mammalian sequences. The tree was constructed based on a Clustal V multiple alignment of the complete sequences for each TNFR and was bootstrapped 1000 times as indicated. Accession numbers of the sequences used for the phylogenetic analysis are as follows: *Homo sapiens* TNFR13C [NP_443177], TNFR13B [NP_036584] and TNFR17 [NP_001183]; *Mus musculus* TNFR13C [NP_082351], TNFR13B [NP_067324] and TNFR17 [NP_035738]; *Oryctolagus cuniculus* TNFR13C [XP_008273381], TNFR13B [XP_008250513] and TNFR17 [XP_008255958]; *Salmo salar* TNFR13C [XP_014059270], TNFR13B [XP_014034414] and TNFR17 [XP_014034440]; *Sinocyclocheilus rhinocerous* TNFR13C [XP_016414908] and TNFR17 [XP_016397935]; *Sinocyclocheilus anshuiensis* TNFR13C [XP_016321777]; *Poecilia latipinna* TNFR13C [XP_014910891]; *Haplochromis burtoni* TNFR13C [XP_005947083]; *Neolamprologus brichardi* TNFR13C [XP_006798027]; *Esox lucius* TNFR13B [XP_010863669]; *Danio rerio* TNFR13B [XP_009304656] and TNFR17 [XP_009304977]; *Cyprinus carpio* TNFR17 [KTF73008].

**Fig 2:**
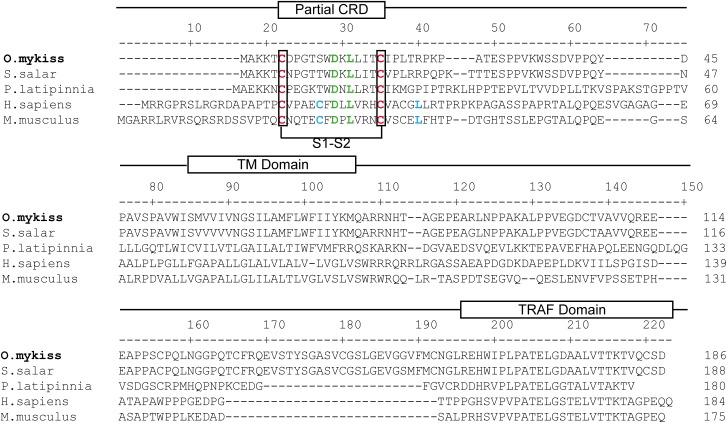
Clustal W alignment of BAFF-R (TNFR13C) from trout (KX894509), salmon (XP_014059270), *P*. *latipinna* (XP_014910891), human (NP_443177) and mouse (NP_082351). The location of the cysteine rich domains (CRD), transmembrane domain (TM) and TRAF binding domain (TRAF domain) are shown above the alignment. Cysteine residues conserved in all species forming disulfide bonds in CRD regions are shown in red and the predicted disulfide bond is indicated with brackets. Residues implicated in receptor binding to BAFF and APRIL are shown in green and those that are known to limit APRIL binding in mammals are indicated in blue.

BAFF-R, BCMA and TACI are all type III transmembrane receptors with a single transmembrane domain, the N terminus on the extracellular side and no signal sequence [37]. After conducting 3´RACE, we obtained a rainbow trout sequence that corresponds to a complete ORF of 561 bp encoding a protein of 186 aa with closest homology to mammalian BAFF-R (Fig 1). The phylogenetic tree clustered all fish and mammalian BAFF-R sequences but included two fish sequences from *Haplochromis burtoni* and *Neolamprologus brichardi* that had been annotated as TNFR17C in GenBank. However, their encoded proteins share the general structure of mammalian BAFF-R and not that of other TNF receptors (Fig 2). TNF receptors are typically organized into multiple cysteine-rich domains (CRDs), each composed of six cysteine residues and three disulfide bonds [38]. These CRDs are involved in interaction with the ligand. In mammals, BAFF-R contains only four cysteine residues in its ligand-binding domain and being the smallest CRD in the TNF receptor family, it has sometimes been referred to as a partial CRD [39]. The cysteine residues within the CRD establish disulfide bridges. Although most CRDs have three disulfide bridges, BAFF-R has been predicted to only establish one disulfide bridge between what have been designated as cysteines 1 and 2, whereas the remaining two cysteines found in the CRD are free. Interestingly, rainbow trout BAFF-R has two conserved cysteines (1 and 2), and therefore could maintain the 1–2 disulfide bridge conserved in all TNF receptors (Fig 2). Some additional residues that condition the binding of BAFF-R to either BAFF or APRIL in mammals are also conserved among fish and mammalian BAFF-R sequences (Fig 2). These include an aspartic acid in position 13 (D26 in human) and a leucine in position 15 (L28 in human), that allow the binding of BAFF-R to both BAFF and APRIL. In contrast, the C24 and the L38 that favor binding to BAFF but are detrimental for APRIL binding in mammals are not conserved in fish [40]. Finally, the COOH-terminal region of BAFF-R was found to be highly conserved between all species, as this is the signaling domain [40]. Although the TRAF-binding signature (P/S/A/T-X-Q/E-E) is not conserved in BAFF-R, this highly-conserved COOH-terminal region is known to bind TRAF3 with high selectivity in mammals [41].

**Fig 3:**
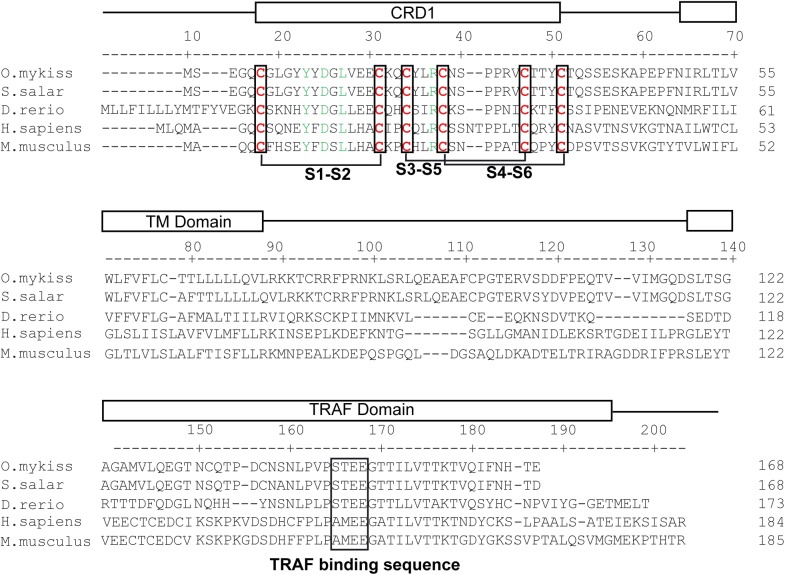
Clustal W alignment of BCMA (TNFR17) from trout (KX894511), salmon (XP_014034440), zebrafish (XP_009304977), human (NP_001183) and mouse (NP_035738). The location of the cysteine rich domains (CRD), transmembrane domain (TM) and TRAF binding domain (TRAF domain) are shown above the alignment. Residues predicted as essential for TRAF binding are boxed (TRAF binding sequence), cysteine residues forming disulfide bonds in CRD regions are shown in red and the predicted disulfide bonds are indicated with brackets. Residues implicated in BAFF and/or APRIL binding are shown in green.

The BCMA sequence identified in rainbow trout includes a complete ORF of 507 bp encoding a protein of 168 aa. Rainbow trout BCMA and other fish BCMA sequences found in the databases clearly clustered with mammalian BCMA sequences (Fig 1). As in mammals, fish BCMA sequences contain one complete CRD composed of six cysteine residues and three disulfide bonds (Fig 3). The sequence also possesses additional conserved residues known to favor BAFF and APRIL binding in mammals. These include a tyrosine in position 11 (Y13 in human), an aspartic acid in position 13 (D15 in human) and a leucine in position 15 (L17 in human). The rainbow trout BCMA sequence also has a conserved arginine in position 25 (R27 in human) which favors APRIL binding and somehow limits the interaction with BAFF [40]. This residue, together with a histidine in position 19 (not present in the fish sequences), accounts for the weak affinity of BAFF for BCMA in mammals. As seen in BAFF-R, the COOH-terminal region of BCMA was also highly conserved between all species, including a TRAF-binding consensus site (P/S/A/T-X-Q/E-E) [40].

**Fig 4:**
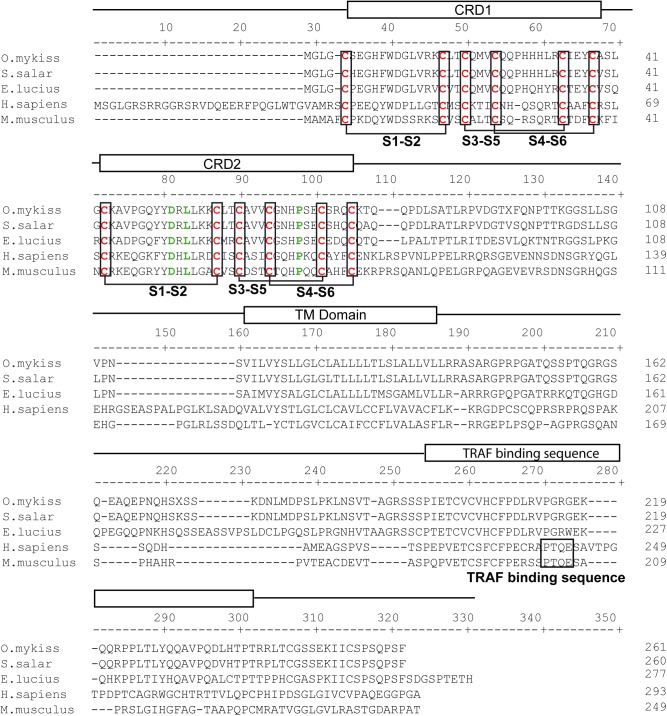
Clustal W alignment of TACI (TNFR13B) from trout (KX894510), salmon (XP_014034414), *Esox lucius* (XP_010863669), human (NP_036584) and mouse (NP_067324). The location of the cysteine rich domains (CRD), transmembrane domain (TM) and TRAF binding domain (TRAF domain) are shown above the alignment. Residues predicted as essential for TRAF binding are boxed (TRAF binding sequence), cysteine residues conserved in all species forming disulfide bonds on each CRD region are shown in red and the predicted disulfide bonds are indicated with brackets. Residues implicated in BAFF and/or APRIL binding are shown in green.

Rainbow trout TACI includes an ORF of 783 bp encoding a protein of 260 aa. The phylogenetic analysis performed clearly grouped rainbow trout TACI with other vertebrate sequences found in GenBank (Fig 1). TACI is characterized by the presence of two CRDs, each composed of six cysteine residues, and this structure has also been conserved in fish (Fig 4). In humans, the first ligand-binding domain has much weaker affinity for BAFF and APRIL than the second one [42]. Consequently, the conserved residues that are known to favor BAFF and APRIL binding are located in CRD2. Many of these residues, such as an aspartic acid in position 52 (D80 in humans), a leucine in position 54 (L82 in humans) and a proline in position 69 (P97 in humans) are conserved in all fish TACI sequences. There is a high degree of homology in the COOH terminal region of all vertebrate TACI sequences, although the consensus TRAF-binding domain found in mammalian TACI sequences is not found in fish TACI sequences.

### Constitutive tissue distribution of BAFF-R, BCMA and TACI

**Fig 5:**
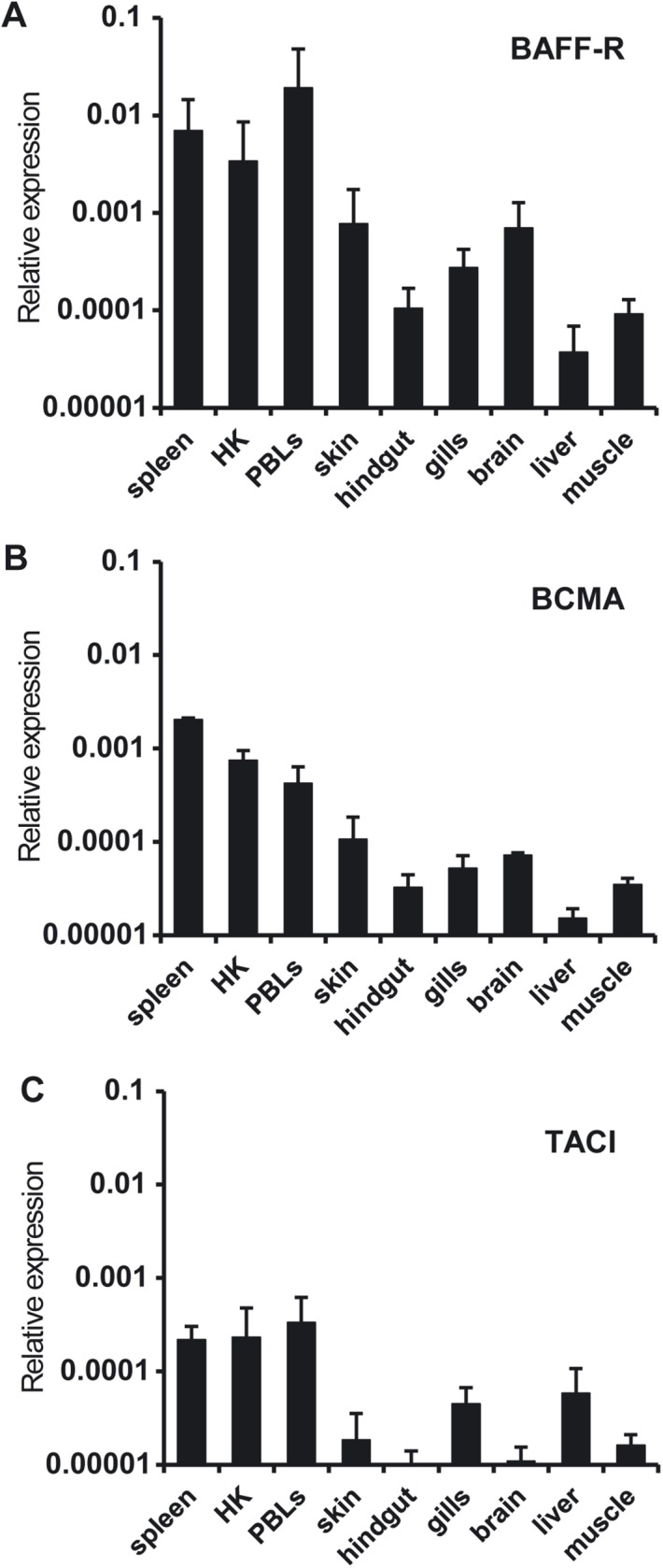
Constitutive levels of transcription of BAFF-R, BCMA and TACI in different tissues. The amount of BAFF-R (A), BCMA (B) and TACI (C) mRNA in PBLs and tissues (spleen, head kidney (HK), skin, hindgut, gills, brain, liver and muscle) from 3 naïve perfused fish was estimated by real time PCR in duplicate samples. Data are shown as the gene expression relative to the expression of endogenous control EF-1α (mean + SD).

We analyzed the levels of constitutive BAFF-R, BCMA and TACI expression in tissues obtained from naïve perfused fish, to avoid contamination of tissues with circulating blood. This is particularly important since we have shown PBLs to constitutively express all three receptors (Fig 5). BAFF-R and BCMA were constitutively transcribed in all tissues analyzed, although higher mRNA levels were observed for both receptors in spleen, PBLs and kidney (Fig 5A and 5B). TACI, on the other hand, was not constitutively transcribed in hindgut and brain (Fig 5C). TACI was expressed in PBLs, spleen and kidney, with low mRNA levels observed in skin, gills, liver and muscle (Fig 5C). In general, BAFF-R mRNA levels were higher than those of the other two receptors, suggesting a more predominant role of BAFF-R in B cell regulation in physiological conditions (Fig 5).

### Transcription of BAFF-R, BCMA and TACI during trout development

**Fig 6:**
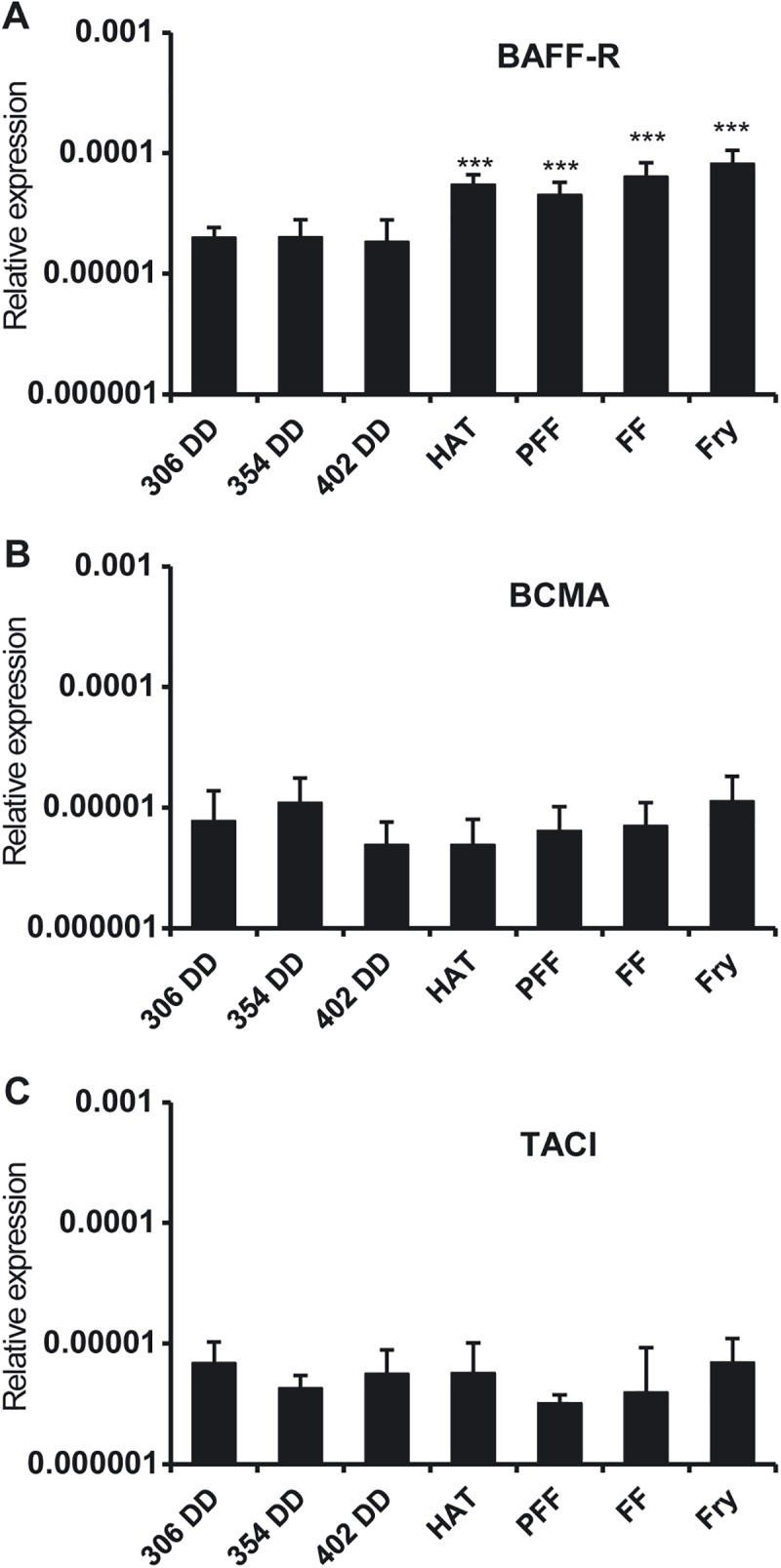
Levels of transcription of BAFF-R, BCMA and TACI in early rainbow trout stages. Transcriptional levels of BAFF-R (A), BCMA (B) and TACI (C) during trout early development at different stages. Data are shown as the gene expression relative to the expression of an endogenous control (EF-1α) (mean + SD, n = 5). DD: degree days; HAT: hatching; PFF: pre-first feeding; FF: first feeding; Fry: 3 weeks post-first feeding. *** Levels of expression significantly different to those observed in samples taken at 640 DD (p < 0.005).

BAFF-R was detected in all the early developmental stages with a significant increase in BAFF-R mRNA levels from hatching (Fig 6A). BCMA and TACI transcription was also detected in all developmental stages, although no significant stage-specific differences in mRNA levels were observed during development (Fig 6B and 6C). Again these results point to a predominant physiological role of BAFF-R.

### BAFF-R, BCMA and TACI transcription in sorted IgM^+^ B cells

**Fig 7:**
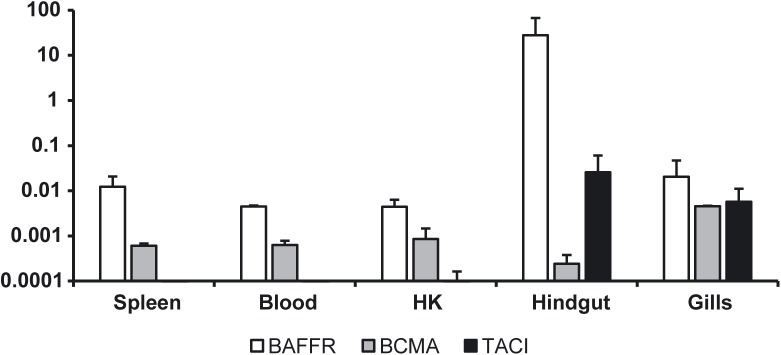
Transcription levels of BAFF-R, BCMA and TACI in B cells. Constitutive levels of transcription of BAFF-R (A), BCMA (B) and TACI (C) in FACS isolated IgM^+^ B cells from different trout tissues were evaluated by real time PCR in duplicate. Results are shown as the gene expression relative to the expression of an endogenous control (EF-1α) (mean + SD, n = 5).

In mammals, BAFF and APRIL receptors are preferentially expressed in B cells, thus we next examined the transcription of BAFF-R, BCMA and TACI in sorted IgM^+^ B cells from different rainbow trout tissues. Spleen, blood and kidney IgM^+^ B cells did not constitutively express TACI transcripts but did express BCMA and BAFF-R (Fig 7). Conversely, IgM^+^ cells from the hindgut had high TACI and BAFF-R transcript levels but low BCMA levels (Fig 7). IgM^+^ B cells from the gills expressed all three receptors at similar levels (Fig 7). These results strongly suggest that in trout IgM^+^ B cells from different sources use these receptors differently to regulate their activation and differentiation processes.

### Effect of PKD on the transcriptional regulation of BAFF, APRIL, BALM and their receptors

**Fig 8:**
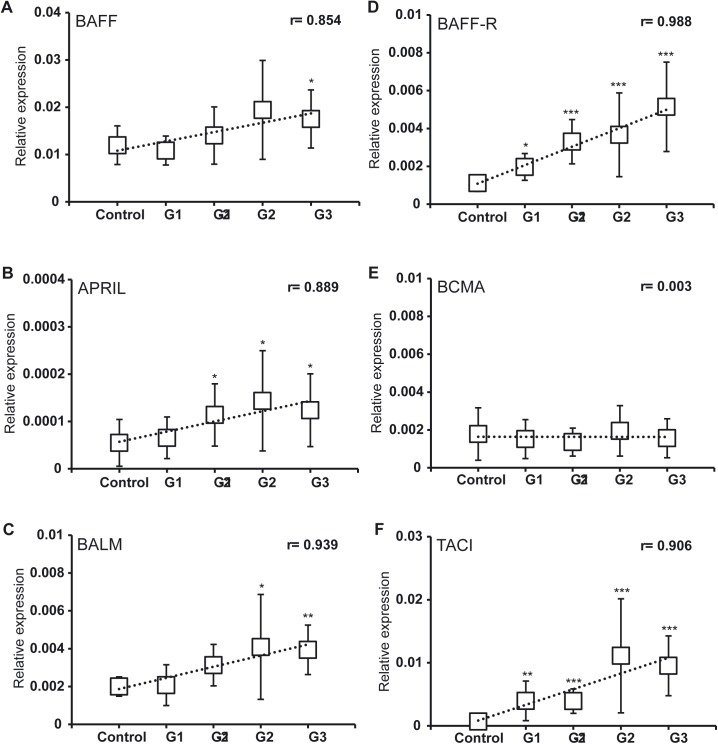
Regulation of BAFF and APRIL subfamily ligands and receptors during PKD infection. Posterior kidney tissue samples were obtained from rainbow trout naturally infected with PKD. Samples were classified according to their kidney swelling grade, as detailed in the Methods. All control fish had a kidney swelling grade of 0 (Control) and samples were named Grade 1 (G1), Grade 1–2 (G1-2), Grade 2 (G2) and Grade 3 (G3). Transcriptional levels of BAFF (A), APRIL (B) and BALM (C) ligands, as well as the BAFF-R (A), BCMA (B) and TACI (C) receptors were evaluated by real time PCR. Results are shown as the gene expression relative to the expression of an endogenous control (EF-1α) (mean ± SD) (Control, n = 10; G1, n = 4, G1-2, n = 7; G2, n = 11, G3, n = 9). Statistical differences between control and infected groups were analyzed with a 2-tailed Student´s *t* test where * *P*<0.05, ** *P*<0.01 and *** *P* < 0.005. A linear regression is also included (dotted line) to show the correlation between the expression of specific genes and the course of the infection. The Pearson product-moment correlation coefficients (r) are also given relative to kidney swelling grade (indicated in the plots).

*In situ* lymphocyte proliferation [43] and hyper-immunoglobulinaemia [24] are two of the most significant traits of clinical PKD in trout. For this reason, we hypothesized that cytokines that are implicated in the control of B cell responses, such as BAFF and APRIL, might be implicated in the pathogenesis of this disease. Thus, we studied the transcription of BAFF, APRIL and BALM as well as the receptors BAFF-R, BCMA and TACI in posterior kidney samples from fish that had been naturally infected with *T*. *bryosalmonae*. Rainbow trout were found to exhibit clinical pathology ranging from grade 1 at low levels to grade 3 in cases of severe/advanced clinical disease. BAFF was significantly upregulated only in grade 3 fish relative to uninfected controls, whereas APRIL and BALM were significantly upregulated in fish graded from 1–2 to 3 and from 2 to 3 respectively (Fig 8). Despite these differences, mRNA levels of all three cytokines strongly correlated with disease progression (*r* values from 0.854 to 0.939) revealing a key role of these three cytokines in PKD pathogenesis in the kidney. Regarding receptor expression, BAFF-R and TACI were both significantly upregulated in infected fish (from grade 1) in comparison to control fish, with a strong correlation apparent between BAFF-R and TACI mRNA levels and disease progression (*r* values 0.988 and 0.906, respectively) (Fig 8D and 8F). In contrast, BCMA transcription was not influenced by parasite infection (Fig 8E), suggesting that this receptor, mostly involved in plasma cell survival [44], is not mediating the effects of BAFF, APRIL and BALM through the course of PKD infection. Despite this, our results suggest that the BAFF, APRIL and BALM signaling axis plays a key role in PKD pathogenesis.

To further understand the situation in the kidney in response to PKD, we analyzed the correlation among ligand and receptor mRNA levels in all kidney samples, as well as the ligands and receptors in relation to fish Igs. Interestingly, BAFF-R mRNA levels correlated significantly with mRNA levels of BAFF, BALM and APRIL (Table 2A). Intriguingly, BCMA mRNA levels only correlated significantly with BAFF (Table 2A), despite the fact that BAFF in mammals has lower affinity than APRIL for BCMA [40]. TACI mRNA levels, in contrast, only correlated significantly with BALM (Table 2A). When correlations among cytokine mRNA levels and levels of transcription of the different Igs were performed, a significant correlation between total IgM mRNA levels and those of the three cytokines was observed, particularly in the case of BALM (Table 2B). BAFF and BALM also correlated significantly with transcripts encoding the secretory forms of IgM and IgT, whereas no correlation was observed with any of the cytokines and IgD (Table 2B). Concerning the receptors, BAFF-R mRNA levels correlated significantly with total IgM, secretory IgM and IgT transcripts, but not with IgD (Table 2C). Despite the fact that BCMA was not significantly induced during PKD progression, there was a significant correlation between IgM levels (both total and secreted) and BCMA transcription (Table 2C). Conversely, no correlation was observed between Ig and TACI expression even though TACI expression was upregulated in parasite exposed fish (Table 2C).

**Table 2:** Correlation of the expression of Ig and BAFF and APRIL subfamily ligands and receptors during the course of PKD infection.

**A**				
	BAFF-R	BCMA	TACI	
BAFF	0.603**	0.426**	0.280	
APRIL	0.602**	0.212	0.178	
BALM	0.641**	0.297	0.356*	
**B**				
	IgM total	IgM sec	IgT	IgD
BAFF	0.407**	0.396**	0.436**	0.128
APRIL	0.396**	0.328	0.302	0.020
BALM	0.708**	0.644**	0.471**	0.242
**C**				
	IgM total	IgM sec	IgT	IgD
BAFF-R	0.593**	0.584**	0.423**	0.261
BCMA	0.512**	0.508**	0.213	0.261
TACI	0.209	0.312	0.123	-0.048

Transcriptional levels of the TNF ligands BAFF, APRIL and BALM, the TNF receptors BAFF-R, BCMA and TACI, and total IgM, secreted IgM (IgM sec), total IgD and total IgT were evaluated at each stage of clinical PKD (grade 1 to 3) by real-time PCR and normalized to the expression of trout EF-1α. Correlations between TNF ligands and TNF receptors (A), TNF ligands and immunoglobulins (B) and TNF receptors and immunoglobulins (C) were studied, and the Pearson product-moment correlation coefficient (r) was calculated for each pair. Statistical differences were analyzed by a 2-tailed Student´s *t* test, and significant differences are shown with numbers in bold, where * *P*<0.05 and ** *P*<0.01.

### Effect of BAFF, APRIL and BALM on the survival of kidney IgM^+^ B cells

**Fig 9:**
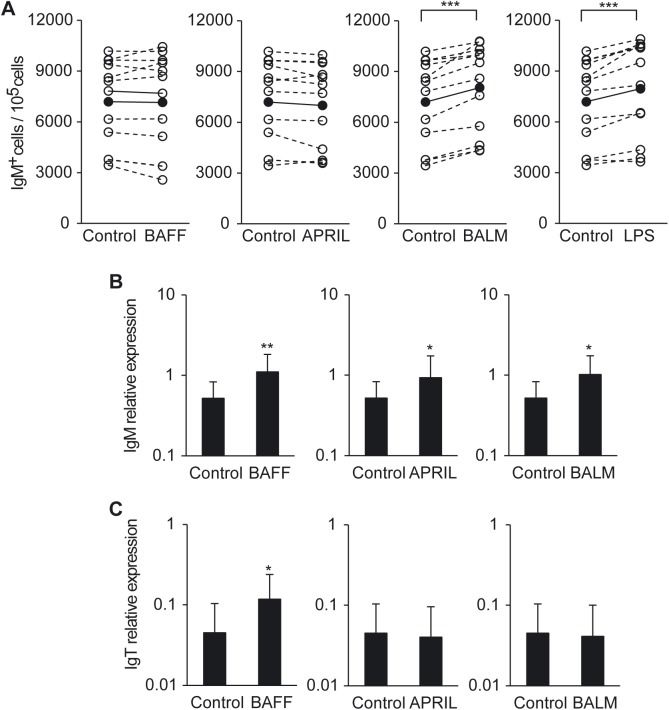
Effect of BAFF, APRIL and BALM on head kidney IgM^+^ B cell survival and immunoglobulin transcription. (A) Head kidney leukocytes were incubated with BAFF (3 μg/ml), APRIL (3 μg/ml), BALM (3 μg/ml), LPS (50 μg/ml) or left unstimulated (control) for 3 days at 20°C. After this time, cells were reacted with an anti-IgM mAb and analyzed by flow cytometry. The total number of IgM^+^ B cells per 10^5^ cells was measured and plotted for each individual fish under control or stimulation conditions. Each animal is represented by a broken line and the mean for each experiment is represented by a solid line (n = 9). (B, C) Head kidney leukocytes were incubated with BAFF (3 μg/ml), APRIL (3 μg/ml), BALM (3 μg/ml) or left unstimulated (control) for 24 h at 20°C. Thereafter RNA was extracted from total leukocytes and the transcription levels of total IgM (B) and total IgT (C) relative to the endogenous control gene EF-1α calculated for each sample and shown as mean + SD (n = 12). Statistical differences were evaluated by a two-tailed Student´s *t* test, where * p < 0.05, ** p < 0.01 and *** p < 0.005.

To gain further insights into the potential impact of BAFF, APRIL and BALM transcriptional upregulation on leukocytes during PKD, we tested the effect of all three cytokines on the survival of IgM^+^ B cells in the kidney. We included LPS in these experiments as a positive control, since LPS has been shown to induce proliferation and increase survival of trout IgM^+^ B cells [45]. Surprisingly, only BALM was capable of significantly promoting the survival of IgM^+^ B cells in the kidney after 3 days of incubation, at similar levels to those induced by LPS (Fig 9A). Thus, we further explored, in cell proliferation assays, the mechanism by which BALM increased IgM^+^ B cell survival, by comparing the effects of this cytokine with those of LPS. Our results demonstrated that BALM was capable of significantly inducing IgM^+^ B cell proliferation (S2 Fig), at levels similar to those triggered by LPS (S2 Fig). Therefore, these proliferative effects likely account for the increase of the total numbers of IgM^+^ B cells observed in BALM-treated cultures.

### Effect of BAFF, APRIL and BALM on the immunoglobulin transcription in kidney

To further investigate the effect that induction of BAFF, APRIL and BALM may have on kidney leukocytes during the course of PKD, we also tested the impact of the three cytokines on the levels of IgM and IgT transcription. In this case, the incubation of kidney cells with the three cytokines significantly increased the levels of transcription of IgM (Fig 9B). These results are in agreement with the positive correlation found between IgM mRNA levels and mRNA levels of BAFF, APRIL and BALM (Table 2). In the case of IgT, however, only BAFF was capable of significantly increasing its transcription levels (Fig 9C).

## Discussion

In the current study, we have identified rainbow trout homologues of the three BAFF and APRIL receptors found in mammals. Although during our database search we identified some additional fish sequences for these receptors, our work constitutes the first in-depth analysis of these receptors in fish. BAFF-R, BCMA and TACI are all type III transmembrane receptors characterized by the N terminus of the protein being on the extracellular side, a single transmembrane domain, absence of a signal peptide, and a COOH terminal region responsible for intracellular signaling [37]. Within the extracellular region, the CRDs are the areas in which ligand binding takes place. Although TNF receptors often have multiple CRDs, BCMA only has one, TACI has two and BAFF-R has only a partial one with four cysteine residues instead of six [37]. Although this general structure was maintained for all three receptors in fish, some important differences were observed in specific residues within the CRD between mammals and fish. In mammals, it has been demonstrated that several conserved residues within the main signaling CRD (CRD1 for BAFF-R and BCMA, and CRD2 in the case of TACI) influence the affinity that each ligand has for each receptor [40]. As a consequence, BAFF binds BAFF-R and TACI with affinities in the nanomolar range, although displaying three orders of magnitude weaker affinity for BCMA, whilst APRIL binds TACI and BCMA with high affinity, but not BAFF-R [40]. However, the two residues known to inhibit the binding of APRIL to BAFF-R in mammals (C24 and L38) are not conserved in fish. Whether this implies that APRIL has the ability to bind to BAFF-R in fish warrants further investigation. This hypothesis is supported by the fact that there is a significant correlation between APRIL and BAFF-R mRNA levels in the kidney of PKD-infected fish. It is, nevertheless, plausible that such positive correlation in gene expression is attributed to both APRIL and BAFF-R correlating with the progression of clinical disease, although TACI is also upregulated during the clinical stages of PKD without correlating to changes in APRIL transcription. In the case of BCMA, amino acid residues H19 and the R27 are known to limit the capacity of BAFF to bind BCMA. In fish, only one of these residues is conserved (R27) with the histidine in position 19 being substituted by a glutamic acid. As before, whether the lack of one of these residues in fish implies a higher affinity of BAFF for BCMA relative to mammals requires further investigation. Nevertheless, during the course of clinical PKD, BCMA transcription correlated significantly with BAFF but not with APRIL or BALM mRNA levels. Importantly, despite the high degree of sequence identity between mammalian and fish TACI sequences in the COOH terminal region, the absence of a TRAF binding consensus site in fish homologues could be indicative of key differences in down-stream intracellular signaling involving TACI in fish relative to mammals.

After sequence verification of rainbow trout BAFF-R, BCMA and TACI, all three receptors were shown to be constitutively expressed in systemic immune tissues (spleen, blood and kidney). In mice, although the expression of BAFF-R protein is low on immature B cells, it increases during B cell maturation and is eventually expressed by all mature B cells [46]. In humans, BAFF-R is widely expressed by all B cells except for bone marrow plasma cells [46]. Rainbow trout BAFF-R was more highly expressed compared to BCMA and TACI, with constitutive expression detected in all tissues analyzed. Furthermore, IgM^+^ B cells from spleen, blood, kidney, hindgut and gills also exhibited high BAFF-R mRNA levels suggesting that this receptor plays an important role in the normal physiology of B cells. BCMA expression, in contrast, is mostly restricted to antibody-producing cells in mammals, as signaling through BCMA is an essential step for plasma cell survival [44]. Rainbow trout BCMA was found to be constitutively expressed in all tissues analyzed and all IgM^+^ B cell populations. It should be noted that although mammalian IgG-producing cells eventually lose the surface Ig expression when they fully differentiate into plasma cells, this does not occur in the case of IgM and IgA plasma cells [47]. Similarly, antibody secreting cells that are differentiating to IgM-secreting plasma cells in rainbow trout have also been shown to retain a functional B cell receptor (BCR) [45]. It is, thus, plausible that some trout IgM^+^ B cell populations that constitutively express BCMA could be plasmablasts/plasma cells. Consistent with mammalian TACI [48], the constitutive nature of fish TACI transcription was much more restricted than that seen with BCMA or BAFF-R, suggesting that TACI is a highly inducible receptor throughout vertebrate phyla. Interestingly, despite its transcription in tissue samples, TACI was not constitutively transcribed by IgM^+^ B cells in spleen, blood or kidney. Thus, the constitutive TACI mRNA levels found in these immune tissues could be attributed to TACI expression in other subsets known to express TACI in specific conditions including monocytes and DCs [49, 50]. In contrast, regardless of the low TACI transcription observed in hindgut and gills, IgM^+^ B cells from these tissues were found to constitutively express TACI, that could be indicative of an important role for TACI in mucosal B cells. Mammalian TACI has been shown to play a crucial role in the differentiation or survival of plasmablasts, particularly those derived from innate B cells [51]. It is also known to be capable of conveying negative signals to B cells through TLR signaling [52]. Thus, in fish, TACI could be contributing towards the maintenance of peripheral tolerance in mucosal sites, a premise that should be addressed in future research.

Given that BAFF-R, BCMA and TACI are expressed in fish B cells and the pathogenesis of PKD being attributed to an apparent lymphoid-driven hyperplastic response and hyperimmunoglobulinaemia [24], we studied the transcription of these receptors and their putative ligands during the course of clinical PKD. BAFF, APRIL, BALM, BAFF-R and TACI were all significantly upregulated in parasite-exposed fish, with transcription levels strongly correlating with PKD progression. Interestingly, BCMA was not transcriptionally influenced by PKD, suggesting a polyclonal or T cell-independent activation of B cells that occurs without the differentiation of activated B cells to plasma cells. Although a complete characterization of the effects of BAFF, APRIL and BALM on fish B cells has not been undertaken to date, some evidence in fish species, such as rohu, imply that increased IgM levels may be attributed to a BAFF-like activity [19], while in some other fish species expansion of B cell populations following BAFF treatment has been reported [11, 16]. It, therefore, seems plausible that BAFF, APRIL and BALM secretion, as well as upregulation of BAFF-R and TACI, are at least partially responsible for the high IgM and IgT levels found in PKD-infected fish [28]. In support of this premise, we have demonstrated that transcription of BAFF, APRIL, BALM, BAFF-R and TACI significantly correlated with total IgM mRNA levels, whereas BAFF, BALM, BAFF-R and TACI transcription correlated with the secreted IgM and IgT mRNA levels. To further confirm a potential role of the BAFF / APRIL axis on PKD pathogenesis, we have studied the effect of the three cytokines on kidney cells. All three cytokines had positive effects on IgM transcription, with BALM also significantly increasing the total number of IgM^+^ B cells through proliferative effects and BAFF being able to augment the transcription of IgT. These results strongly suggest that the synthesis of these cytokines during the course of PKD accounts, at least partially, for the increased immunoglobulin secretion observed in response to the parasite. Our experiments showing the lymphoproliferative effects of BALM and its capacity to upregulate IgM transcription provide the first evidences of a functional role for BALM, an ancient precursor of mammalian BAFF and APRIL.

Treatments with chemicals such as malachite green and fumigillin are effective against PKD, but are not licensable due to their toxicity effects in humans [53, 54]. The data presented here provides a fascinating insight into potential immune therapies that could be developed in the future to control PKD pathogenesis in the absence of an effective vaccine. Increased serum levels of BAFF and/or APRIL are often found in patients with autoimmune diseases, including systemic lupus erythematosus (SLE) or rheumatoid arthritis [55]. The appreciation that BAFF overexpression induced in murine models causes SLE and that BAFF inhibition delays SLE onset has encouraged the development of therapeutic agents for inhibiting BAFF, APRIL, or their receptors, as treatments for SLE and other autoimmune disorders [56]. These blocking strategies include a monoclonal antibody against soluble BAFF, antibodies recognizing both soluble and membrane BAFF, and a TACI-Ig fusion protein. Such strategies to curtail human autoimmune disorders have been the subject of recent clinical trials [57]. In this context, a similar approach for sequestering BAFF, APRIL, BALM or blocking their receptors could be employed for the control of PKD in fish given the fact that fish infected with *T*. *bryosalmonae* exhibit a chronic immune disorder that in some ways resembles an autoimmune disease.

In summary, we have identified for the first time in fish the three BAFF and APRIL receptors known in mammals, namely TACI, BCMA and BAFF-R. Additionally, we report the regulation of BAFF-R and TACI and their potential ligands, BAFF, APRIL and BALM during the course of PKD in rainbow trout, as well as the correlation between the transcription of these genes and Igs. These results, together with those obtained from functional studies that point to a role for these cytokines in B cell function strongly suggest that activation of the BAFF / APRIL axis contributes to the hyper-immunoglobulinaemia and B cell dysregulation that characterizes this disease. Given the lack of effective vaccines to prevent PKD, the immunoprophylactic potential of BAFF / APRIL axis modulation could be explored in the future as an alternative disease control strategy to vaccine development.

## Supporting information

S1 Fig Gating strategy for FACS isolation of IgM^+^ B cells. Flow cytometry analysis of rainbow trout leukocytes isolated from trout tissues (spleen, blood, head kidney, PBLs, hindgut and gills) and labeled with an anti-IgM mAb. For each individual tissue, FSC/SSC profiles including a defined gate for lymphoid cells are shown (top row). IgM staining within the lymphoid gate is also shown (bottom row dot plots). Lymphoid IgM^+^ (lower right corner gate) cells were FACS isolated as described in the Methods. A representative experiment out of 3 independent assays is shown (n = 9).
(PDF)

S2 Fig Rainbow trout BALM possesses lymphoproliferative effects. To test the effect of BALM on IgM^+^ B cell proliferation, head kidney leukocytes were incubated with BALM (3 μg/ml), LPS (50 μg/ml), or left unstimulated (control) for 4 days at 20°C. After this time, cells were labeled with 10mM EdU and incubated for a further 2 h. Then, the cells were labelled with an anti-IgM mAb, and treated for cell proliferation assays, as described in the Methods. The percentage of proliferating (EdU^+^) IgM^+^ B cells was then determined by flow cytometry analysis. Quantification of the proliferating IgM^+^ populations is shown as mean + SD (left, n = 6), together with a representative dot plot of the flow cytometry analysis (right). Number of proliferating IgM^+^ cells are also indicated within the dot plots. Statistical differences were evaluated by a two-tailed Student´s *t* test, where ** p ≤ 0.01 and *** p ≤ 0.005.
(PDF)
