# Supplementary material for: Characterization of BAFF and APRIL subfamily receptors in rainbow trout (Oncorhynchus mykiss). Potential role of the BAFF / APRIL axis in the pathogenesis of proliferative kidney disease
Source: PLoS One. 2017 Mar 21;12(3):e0174249. doi: 10.1371/journal.pone.0174249 (PMC5360319; doi:10.1371/journal.pone.0174249)
Supplement: S1 Fig — Flow cytometry analysis of rainbow trout leukocytes isolated from trout tissues (spleen, blood, head kidney, PBLs, hindgut and gills) and labeled with an anti-IgM mAb. For each individual tissue, FSC/SSC profiles including a defined gate for lymphoid cells are shown (top row). IgM staining within the lymphoid gate is also shown (bottom row dot plots). Lymphoid IgM+ (lower right corner gate) cells were FACS isolated as described in the Methods. A representative experiment out of 3 independent assays is shown (n = 9). (PDF) [file pone.0174249.s001.pdf]

S1 Fig

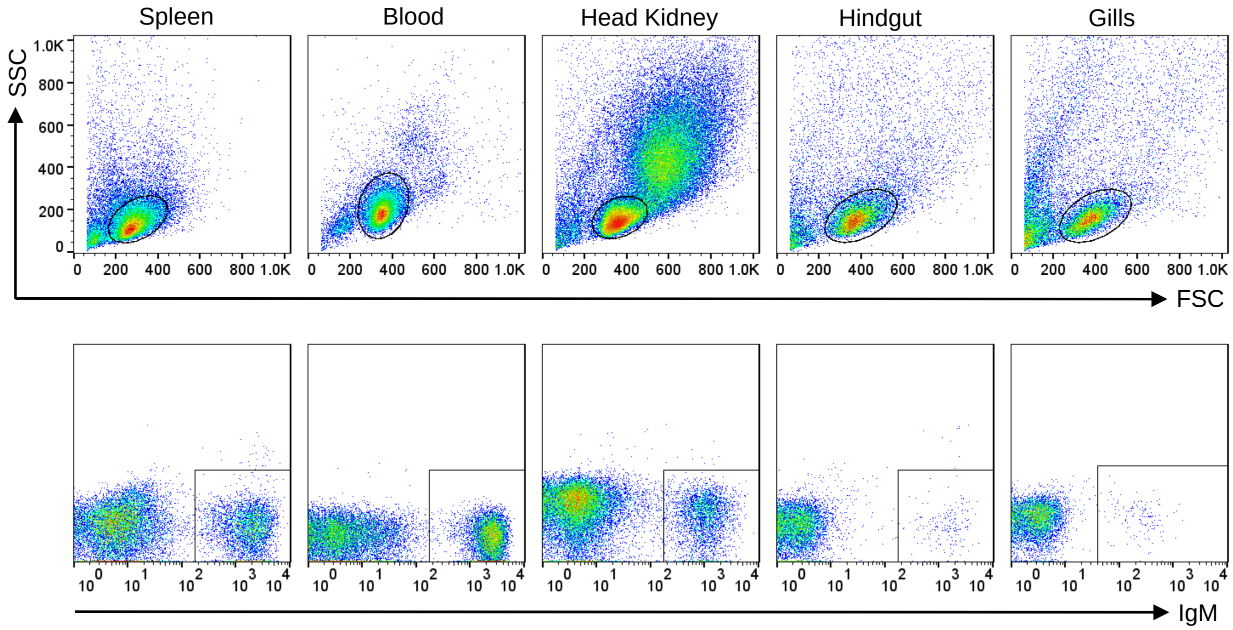

S1 Figure. Gating strategy for FACS isolation of IgM$^+$ B cells. Flow cytometry analysis of rainbow trout leukocytes isolated from trout tissues (spleen, blood, head kidney, PBLs, hindgut and gills) and stained with an anti-IgM mAb. For each individual tissue, FSC/SSC profiles including a defined gate for lymphoid cells are shown (top row). IgM staining within the lymphoid gate is also shown (bottom row dot plots). Lymphoid IgM$^+$ (lower right corner gate) cells were FACS isolated as described in Methods. A representative experiment out of 3 independent assays is shown (n=9).
