# Supplementary material for: Characterization of BAFF and APRIL subfamily receptors in rainbow trout (Oncorhynchus mykiss). Potential role of the BAFF / APRIL axis in the pathogenesis of proliferative kidney disease
Source: PLoS One. 2017 Mar 21;12(3):e0174249. doi: 10.1371/journal.pone.0174249 (PMC5360319; doi:10.1371/journal.pone.0174249)
Supplement: S2 Fig — To test the effect of BALM on IgM+ B cell proliferation, head kidney leukocytes were incubated with BALM (3 μg/ml), LPS (50 μg/ml), or left unstimulated (control) for 4 days at 20°C. After this time, cells were labeled with 10mM EdU and incubated for a further 2 h. Then, the cells were labelled with an anti-IgM mAb, and treated for cell proliferation assays, as described in the Methods. The percentage of proliferating (EdU+) IgM+ B cells was then determined by flow cytometry analysis. Quantification of the proliferating IgM+ populations is shown as mean + SD (left, n = 6), together with a representative dot plot of the flow cytometry analysis (right). Number of proliferating IgM+ cells are also indicated within the dot plots. Statistical differences were evaluated by a two-tailed Student´s t test, where ** p ≤ 0.01 and *** p ≤ 0.005. (PDF) [file pone.0174249.s002.pdf]

S2 Fig

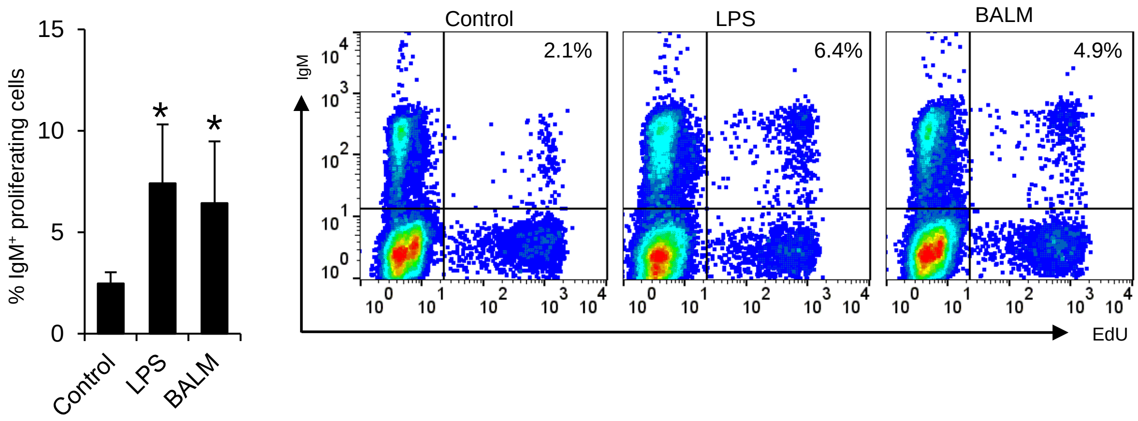

S2 Figure. Rainbow trout BALM possesses lymphoproliferative effects. To test the effect of BALM on IgM$^+$ B cell proliferation, head kidney leukocytes were incubated with BALM (3 µg/ml), LPS (50 µg/ml), or left unstimulated (control) for 4 days at 20°C. After this time, cells were labeled with 10µM EdU and incubated for a further 2 h. Then, the cells were labelled with an anti-IgM mAb, and treated for cell proliferation assays, as described in Methods. The percentage of proliferating (EdU$^+$) IgM$^+$ B cells was then determined by flow cytometry analysis. Quantification of the proliferating IgM$^+$ populations is shown as mean + SD (left, n=6), together with a representative dot plot of the flow cytometry analysis (right). Number of proliferating IgM$^+$ cells are also indicated within the dot plots. Statistical differences were evaluated by a two-tailed Student´s *t* test, where * means $p \leq 0.05$.
